# Sipa1l1 is an early biomarker of liver fibrosis in CCl_4_-treated rats

**DOI:** 10.1242/bio.018887

**Published:** 2016-05-26

**Authors:** Santiago Marfà, Manuel Morales-Ruiz, Denise Oró, Jordi Ribera, Guillermo Fernández-Varo, Wladimiro Jiménez

**Affiliations:** 1 Biochemistry and Molecular Genetics Service, Hospital Clinic, Centro de Investigación Biomédica en Red de Enfermedades Hepáticas y Digestivas (CIBEREHD), Institut d'Investigacions Biomèdiques August Pi i Sunyer (IDIBAPS), 08036 Barcelona, Spain; 2 Departament de Biomedicina, University of Barcelona, 08036 Barcelona, Spain

**Keywords:** Liver fibrosis, Biomarker, Proteomics, SIPA1L1, E6TP1

## Abstract

At present, several procedures are used for staging liver fibrosis. However, these methods may involve clinical complications and/or present diagnostic uncertainty mainly in the early stages of the disease. Thus, this study was designed to unveil new non-invasive biomarkers of liver fibrosis in an *in vivo* model of fibrosis/cirrhosis induction by CCl_4_ inhalation by using a label-free quantitative LC-MS/MS approach. We analyzed 94 serum samples from adult Wistar rats with different degrees of liver fibrosis and 36 control rats. Firstly, serum samples from 18 CCl_4_-treated rats were clustered into three different groups according to the severity of hepatic and the serum proteome was characterized by label-free LC-MS/MS. Furthermore, three different pooled serum samples obtained from 16 control Wistar rats were also analyzed. Based on the proteomic data obtained, we performed a multivariate analysis which displayed three main cell signaling pathways altered in fibrosis. In cirrhosis, more biological imbalances were detected as well as multi-organ alterations. In addition, hemopexin and signal-induced proliferation-associated 1 like 1 (SIPA1L1) were selected as potential serum markers of liver fibrogenesis among all the analyzed proteins. The results were validated by ELISA in an independent group of 76 fibrotic/cirrhotic rats and 20 controls which confirmed SIPA1L1 as a potential non-invasive biomarker of liver fibrosis. In particular, SIPA1L1 showed a clear diminution in serum samples from fibrotic/cirrhotic rats and a great accuracy at identifying early fibrotic stages. In conclusion, the proteomic analysis of serum samples from CCl_4_-treated rats has enabled the identification of SIPA1L1 as a non-invasive marker of early liver fibrosis.

## Abstract (precis)

**Summary:** Proteomic analysis of serum samples from fibrotic and cirrhotic rats revealed a reduction in SIPA1L1 concentration with potential use as a marker for early liver fibrosis diagnosis.

## INTRODUCTION

The identification of non-invasive markers of fibroproliferative processes has been subject of considerable investigation during the last decade (Fernandez-Varo and Jimenez, 2011; Martinez et al., 2011a). The huge burden of these pathologies and the availability of next-generation antifibrotic drugs allowing early therapeutic interventions have been major drivers in achieving this goal. Despite these efforts, however, the challenge remains elusive. The natural history of most liver diseases is probably among the major reasons accounting for this. Liver diseases are usually an insidious process that may evolve for long periods of time and may develop without evident clinical manifestations during the first years of evolution after the onset of injury (Hernandez-Gea and Friedman, 2011; Hannivoort et al., 2012). Under this scenario, the search for fibroproliferative processes has become endless. In the current investigation we attempted to circumvent this difficulty by unveiling circulating biomarkers of liver fibrosis in rats with carbon tetrachloride (CCl_4_)-induced fibrosis. In this experimental model the time to detect histological signals of hepatic healing is considerably shortened and it also allows the collection of tissue samples, blood and clinical data at different points of disease evolution (Clària and Jiménez, 2005). This experimental approach was combined with a label-free liquid chromatography-tandem mass spectrometry (LC-MS/MS) workflow in a large set of serum samples obtained from fibrotic rats and healthy animals. Label-free quantitative LC-MS/MS is a powerful method for identifying and quantifying proteins in complex samples (Altelaar et al., 2013; Sandin et al., 2015). This pilot study was initially performed for biomarker candidate selection and was followed up with enzyme-linked immunosorbent assay (ELISA) validation of two biomarkers in a larger sample cohort. Following this discovery protocol, we were able to uncover signal-induced proliferation-associated 1 like 1 (SIPA1L1) as a new non-previously described early biomarker of liver fibrosis in CCl_4_-treated rats, thus providing scientific rational to design clinical studies assessing the diagnostic and/or prognostic utility of SIPA1L1 in patients with liver fibrosis.

## RESULTS

On histological examination, fibrotic lesions evolved from a weak deposition basically in the portal area (mild fibrosis) to several thicker septa which resulted from more prolonged exposure to CCl_4_ (severe fibrosis). Finally, most of the animals exposed to CCl_4_ for longer periods of time developed cirrhosis, and the histological analysis displayed the formation of regenerative nodules of liver parenchyma separated by fibrotic septa (Fig. S1). Control CCl_4_ non-treated rats displayed no appreciable alterations in liver histology and an almost negligible amount of fibrous tissue. Table 1 shows serum electrolytes and the biochemical tests of liver function in control and CCl_4_-treated rats included in the training group. As expected, fibrotic and cirrhotic rats included in this group had important and progressive alterations in liver-function tests, being more pronounced in cirrhotic animals.

**Table 1.**
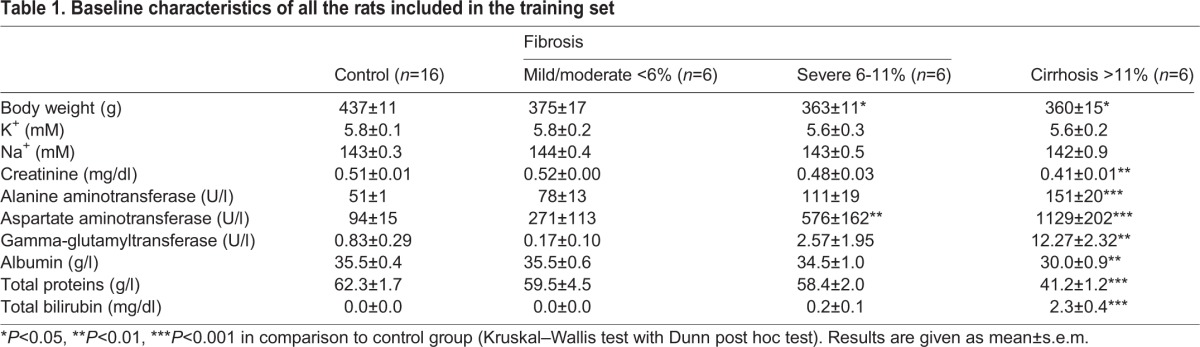
**Baseline characteristics of all the rats included in the training set**

### Proteomic signature of CCl_4_-treated rats and signaling pathway analysis

**Fig. 1.**
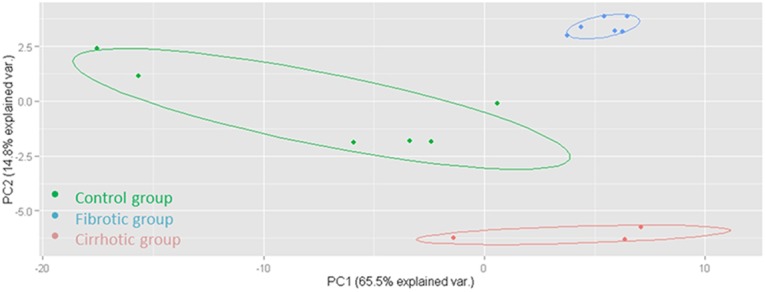
**Multivariate data analysis of serum samples from CCl_4_-treated rats and the control group.** PCA of the serum proteomic data obtained from CCl_4_-treated and control rats. Only the first two principal components were plotted. The score plot displayed a clear separation between the control, fibrotic and cirrhotic groups, despite no differences being observed between mild/moderate fibrosis and severe fibrosis.

**Fig. 2.**
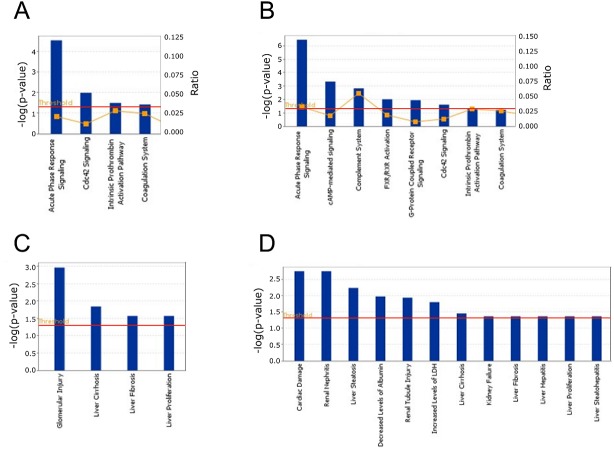
**Signaling pathway analysis.** The comparisons of the cell signaling pathways (A,B) and the pathological mechanisms (C,D) occurring in fibrotic (A,C) and cirrhotic rats (B,D) were modeled using the Ingenuity Pathways Knowledge Base (IPA). The proteomic results obtained in control, fibrotic and cirrhotic rats were compared with global molecular networks using the Fisher's Exact Test. The resulting *P*-values were adjusted for multiple comparisons using the Benjamini and Hochberg's method to control the false discovery rate. After multitest adjustment, differences were considered to be significant at *P<*0.05. The red line across the cell signaling graphs delimits the point where *P=*0.05. In addition, the significance of the association was measured on the basis of the ratio of the number of targets from the data set that map to the pathway divided by the total number of targets that are included in the canonical pathway (yellow line and right axis, top panels). Only proteomic changes larger than twofold were included in the pathway analysis.

To determine if there is a clustering of observations suggesting an underlying multivariate pattern of proteins, a principal component analysis (PCA) was performed. The 45 most discriminatory proteins, as reflected by the highest statistical significance when compared to control samples, were included in the analysis (Table S1). No differences were observed among fibrotic rats regardless of the presentation of mild/moderate or severe fibrosis. However the scatter plot visually conveys the separation of fibrotic, cirrhotic and control rats with no overlapping among samples (Fig. 1). Afterwards, two quantitative signaling pathway analyses were performed based on all the proteins identified and quantified (Fig. 2). Clear alterations in the coagulation cascade (coagulation system and intrinsic prothrombin activation pathway) as well as variations in the acute phase response signaling were found. The regulation of proliferative signaling was also affected in the fibrotic group (Fig. 2A). As expected, the cirrhosis signaling pathway analysis showed more anomalies. Apart from those observed in fibrosis, other alterations such as the G-protein coupled receptor signaling pathway or the cAMP-mediated signaling were also statistically significant, among others (Fig. 2B). Finally, several pathological mechanisms were detected in both the fibrotic (Fig. 2C) and cirrhotic groups (Fig. 2D), mainly related to the liver disease. Of note was that pathways related to cardiac damage or renal dysfunction were also affected in the group of cirrhotic rats thus reinforcing the confidence of the performed analysis.

### Hemopexin and SIPA1L1 as potential serum biomarkers for fibrosis and cirrhosis detection

**Fig. 3.**
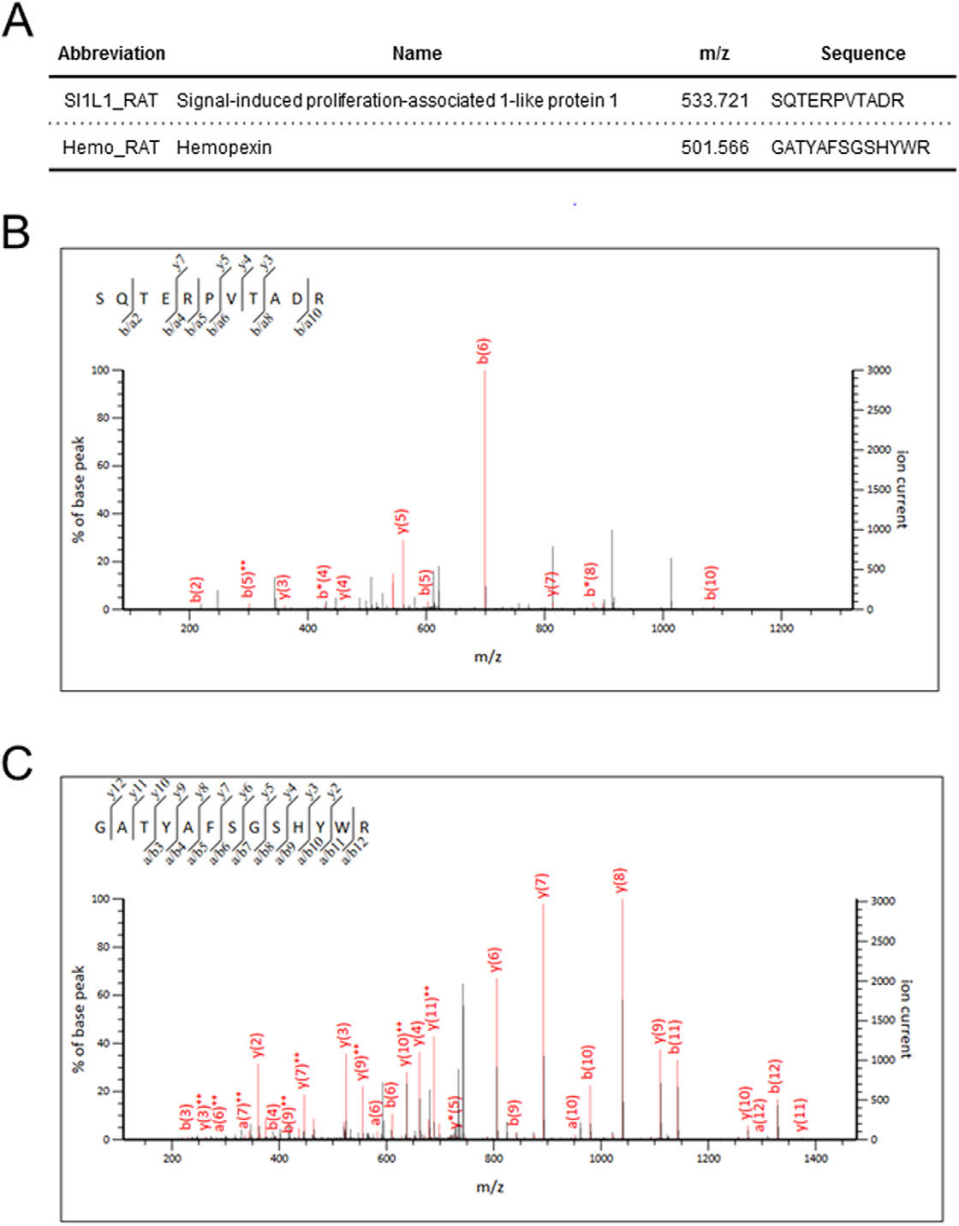
**MS/MS analysis of the SIPA1L1 and hemopexin peptides identified.** (A) Amino acid sequencing of SIPA1L1 and hemopexin. (B,C) Representative MS/MS spectra of the peptides identified from SIPA1L1 (B) and hemopexin (C).

The ten different protein peaks showing the most statistically significant expression in samples from CCl_4_-treated rats as compared to controls were selected. Among these, four were specifically detected in samples from fibrotic animals whereas six were exclusively found in cirrhotic rats (Table 2). Only proteins showing at least a twofold change in expression were further considered for subsequent analysis. Moreover, high-abundant serum proteins and proteins not very conserved between rats and humans were also excluded. According to this data processing strategy, only SIPA1L1 and hemopexin were selected. In particular, SIPA1L1 was down expressed in fibrosis whereas hemopexin was increased in cirrhosis. Representative MS/MS spectra from the SIPA1L1 and hemopexin peptides are shown in Fig. 3.

**Table 2.**
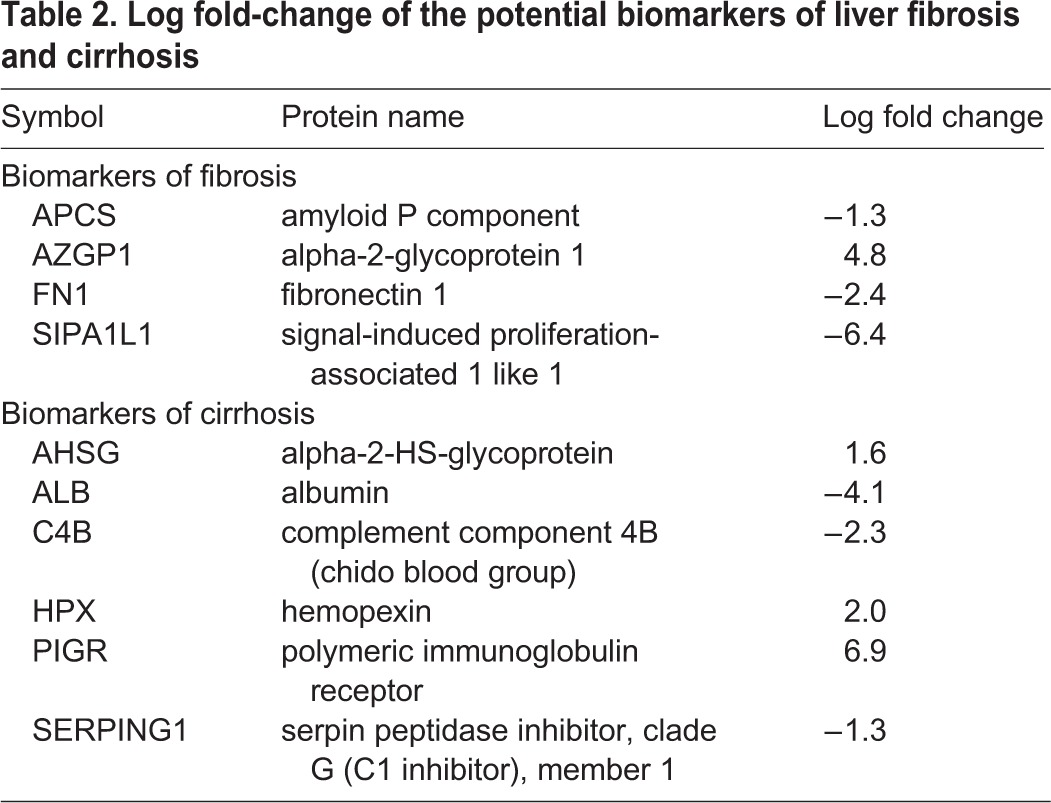
**Log fold-change of the potential biomarkers of liver fibrosis and cirrhosis**

### Biomarker assessment in the validation group

To confirm that SIPA1L1 and hemopexin were potential biomarkers for the detection of liver fibrosis and cirrhosis respectively, both candidates were measured by ELISA in a large validation group of CCl_4_-treated rats with different degrees of liver fibrosis. Table 3 shows systemic and portal hemodynamics, serum electrolytes and standard parameters of liver function in all animals included in the validation protocol. Paralleling the results found in the training protocols, CCl_4_-treated rats showed a progressive deterioration of hepatic enzymes and liver function as fibrosis evolved, which was also associated with a gradual decrease in mean arterial pressure (MAP) and increased portal pressure (PP).

**Table 3.**
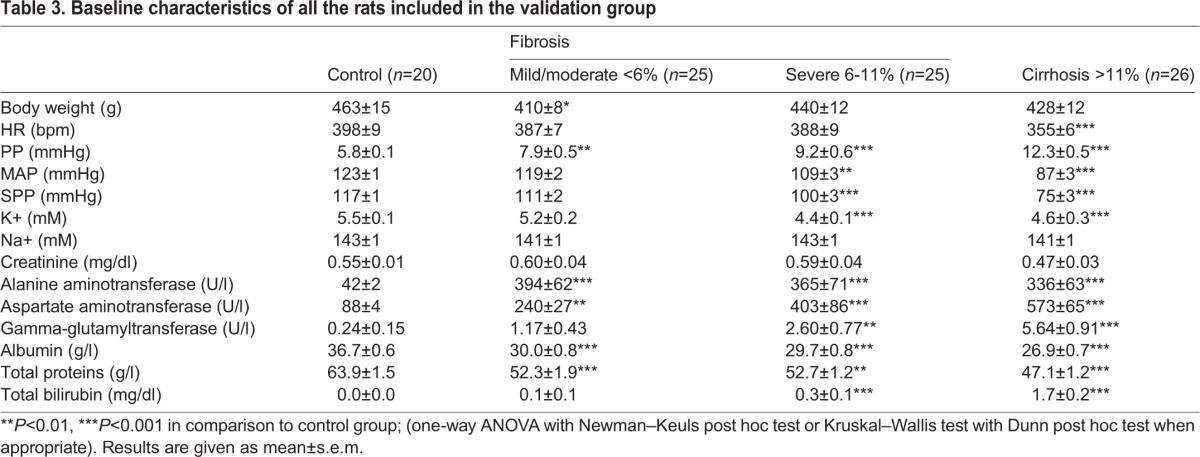
**Baseline characteristics of all the rats included in the validation group**

**Fig. 4.**
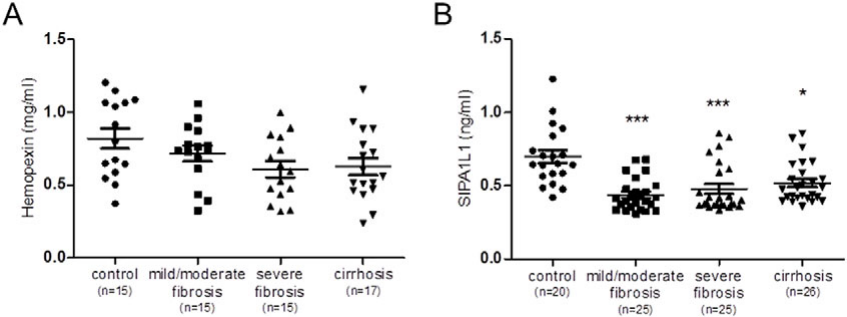
**Serum concentration of hemopexin and SIPA1L1 in CCl_4_-treated rats.** (A) Serum hemopexin values obtained from control (*n*=15), mild/moderate (*n*=15), severe fibrotic (*n*=15) and cirrhotic rats (*n*=17). One-way ANOVA with Newman–Keuls post hoc test was used to evaluate dissimilarities between groups. No differences were found between groups. (B) SIPA1L1 serum levels in control rats (*n*=20), mild/moderate (*n*=25) and severe fibrotic rats (*n*=25) and cirrhotic (*n*=26). Differences between groups were evaluated by the Kruskal–Wallis test with the Dunn post hoc test. *P<0.05, ***P<0.001 in comparison to the control group.

As shown in Fig. 4A, no differences were detected in the circulating levels of hemopexin between control and CCl_4_-treated rats, including cirrhotic animals. These results, therefore, failed to confirm hemopexin as a non-invasive biomarker of liver fibrosis/cirrhosis in rats. In contrast, CCl_4_-treated rats with mild fibrosis showed a significant reduction in the serum concentration of SIPA1L1 as compared to controls. These results were coincident to those found in the label free quantitative LC-MS/MS analysis. Furthermore, this diminution was also observed in rats with severe fibrosis and cirrhosis (Fig. 4B).

### Accuracy of SIPA1L1 and hemopexin to detect early stages of hepatic fibrosis and cirrhosis, respectively

**Fig. 5.**
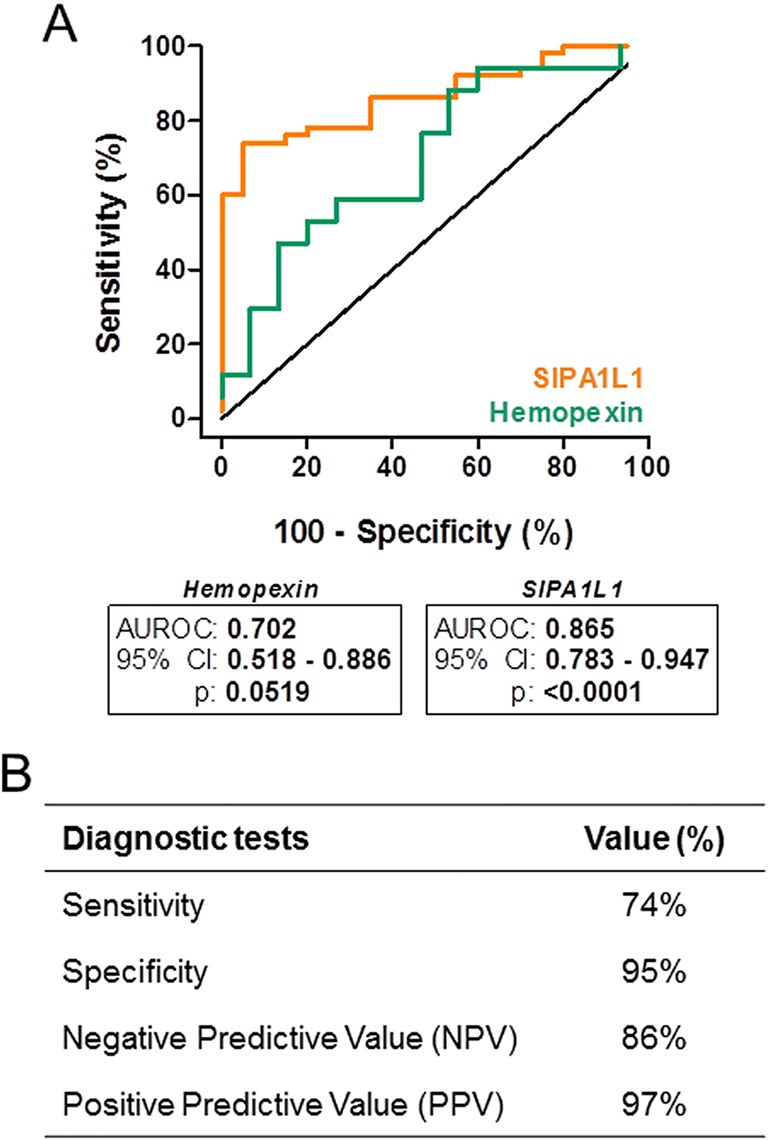
**Diagnostic accuracy of hemopexin and SIPA1L1 and diagnostic tests of SIPA1L1.** (A) Diagnostic accuracy of hemopexin and SIPA1L1 to differentiate cirrhosis and fibrosis, respectively. When using the hemopexin serum concentration, the AUROC was 0.702 but not statistically significant (*P*>0.05). The AUROC obtained from SIPA1L1 serum concentration was 0.865 and statistically significant (*P*<0.0001). (B) Several diagnostic tests (Sensitivity, Specificity, NPV and PPV) were performed in SIPA1L1 after determining 475.5 pg/ml as the optimal cutoff.

The results for the accuracy of SIPA1L1 and hemopexin in fibrotic and cirrhotic rats are presented as receiver-operating characteristic (ROC) curves in Fig. 5A. Animals belonging to the fibrotic groups (*n*=50) were considered in the analysis for SIPA1L1, whereas only cirrhotic rats were considered at assessing hemopexin (*n*=17). The ROC of SIPA1L1 showed an excellent diagnostic accuracy to discriminate rats with fibrosis from control animals [area under the ROC (AUROC): 0.865, *P*<0.0001]. In contrast, the ROC of hemopexin to discriminate cirrhotic from control rats displayed a considerably lower diagnostic efficacy (AUROC: 0.702), which lacked statistical significance. In addition, a serum concentration of SIPAL1L1 of 475.5 pg/ml was selected as the optimal cutoff value to differentiate normal from fibrotic animals. This estimation was based on the maximum value of the likelihood ratio, which minimizes the number of false positive and false negative cases. Above this cutoff, 86% of rats did not show significant fibrosis. Below this cutoff, 97% of rats had fibrosis. In addition, we correctly classified 74% of the animals with fibrosis. Finally, the specificity was also determined and reached the 95% (Fig. 5B).

## DISCUSSION

Early detection of fibrosis progression is an essential step for preventing future clinical complications in patients with chronic liver disease. At present, liver biopsy is still the gold standard procedure to assess hepatic fibrosis (Fernandez-Varo and Jimenez, 2011; Castera and Pinzani, 2010), but the risk of clinical complications and sampling errors are some of the most remarkable limitations (Martinez et al., 2011a; Cadranel et al., 2000; West and Card, 2010). In this context, new non-invasive biomarkers have recently appeared as potential alternatives to liver biopsy. However, early detection of hepatic fibrosis remains an open challenge, as the diagnostic capacity of many circulating markers and algorithms are not as accurate in the early and mild stages of liver fibrosis in comparison to advanced fibrosis (Lichtinghagen et al., 2013; Martinez et al., 2011b). In most cases the slow progression of the disease emerges as one of the main obstacles for discovering specific biomarkers of early fibrosis.

In the present study this difficulty was circumvented using a strategy for identifying biomarkers of liver fibrosis in an experimental model of rats with CCl_4_-induced fibrosis and cirrhosis that closely reproduces the histological, hemodynamic, biochemical and renal disturbances that patients with liver disease develop. Actually, previous investigations from our laboratory and others have demonstrated that these animals have a gradually increased hepatic deposition of collagen fibers which is associated with a progressive derangement in systemic and splanchnich hemodynamics, altered serum concentrations of liver and renal function tests and marked sodium and water retention that results in ascites formation (Clària and Jiménez, 2005). This sequence of events is very similar to that observed in human cirrhosis and lays the foundation to use this model to better understand the pathophisiological mechanisms involved in these phenomena, as well as to evaluate the therapeutic utility of new drugs to ultimately be used in patients with liver disease (Fernández-Varo et al., 2016; Oró et al., 2016). The results found in the training and validation groups further confirm this contention. In fact, collagen deposition in the CCl_4_-treated rats ranged between 1.9% and 26.3% according to the time submitted to the fibrosis induction protocol. We also observed a progressively decreased MAP and increased PP. Moreover, low serum concentrations of albumin and marked activation of hepatic enzymes were also noted. Finally, ascites was detected in some of the animals with higher hepatic collagen content. All these data confirm the suitability of the experimental model to uncover new biomarkers of liver fibrosis.

Two-dimensional gel electrophoresis (2-DE) has been the most widely used proteomic method for comparing protein expression profiles between normal and pathological conditions (Srinivas et al., 2001; Adam et al., 2001). However, the 2-DE approach has several disadvantages, including the difficulty in separating proteins with extremes in molecular weight (<10 kDa or >200 kDa) and in isoelectric points (<4 or >10) as well as complications for resolving hydrophobic proteins or even detecting low abundance proteins (Rabilloud, 2002; Braun et al., 2007). To overcome these drawbacks, label-free LC-MS/MS has become indispensable in the proteome analysis of several diseases (Altelaar et al., 2013) including kidney chronic allograft dysfunction and hematological malignances (Quintana et al., 2009; Casado et al., 2013). Furthermore, this technique has emerged as a promising alternative for biomarker discovery (Sandin et al., 2015). Compared to the protein-labeling approaches, label-free quantitative methods allow individual analysis of each sample, enabling the study of a large number of specimens for each experiment due to the absence of labeling restrictions. Data analysis, encompassing protein identification and quantification provides a general overview of all the proteomic data obtained from the MS assessment. In the current investigation multivariate PCA was adopted to explore and visualize a protein biomarker fingerprint associated with hepatic fibrosis. Following the identification of the 45 peaks showing higher statistical difference between fibrotic and control samples in the definition/training groups, the PCA was able to distinguish a different protein signature for fibrotic, cirrhotic and control samples. Interestingly enough, this proteomic analysis did not establish clear differences between rats with mild/moderate or severe fibrosis. A subsequent analysis performed by clustering all the proteomic data into the different cell signaling pathways pointed out three major altered signaling pathways in the fibrotic animals, i.e. the acute phase response, the coagulation cascade and mechanisms involved in cell proliferation. As anticipated, the amount of deregulated signaling pathways in cirrhotic animals was higher and affected other organs than the liver, such as kidney and heart. These findings are in concordance with the most characteristic pathological features already described in patients with different degrees of alteration in hepatic architecture (Mercer and Chambers, 2013; Betrosian et al., 2007; Møller and Henriksen, 2002), thus further validating the usefulness of CCl_4_-treated rats in biomarker discovery for liver disease.

Label free LC-MS/MS proteomic analysis resulted in the identification of two non-invasive independent circulating proteins, SIPA1L1 and hemopexin, biomarkers of fibrosis and cirrhosis, respectively. Three isoforms of SIPA1L1 of approximately 200 kDa have been described (Gao et al., 1999). At present, SIPA1L1 is known to be a GTPase-activating protein (GAP) as it possesses a region of homology with GAP domains (Gao et al., 1999). On the other hand, SIPA1L1 has also been described as a potential tumor suppressor (Gao et al., 1999) since it is located on a region in chromosome 14 which has been reported to exhibit a loss of heterozygosity in malignant meningiomas (Menon et al., 1997). Thus, it seems that its inactivation contributes to malignant transformations. In the current investigation, we observed a significant diminution of SIPA1L1 serum levels in mild/moderate fibrosis and this reduction was maintained over the fibrotic and cirrhotic groups. Furthermore, SIPA1L1 amino acid sequence is highly conserved between humans and rats. In particular, and according to the GeneCards database, the similarity reaches the 95.34% of coincidence. Therefore, it is feasible to think that the results obtained from the *in vivo* model could be translated to human liver fibrosis, since proteins with very similar amino acid composition and sequence often implies similar functions (Alberts et al., 2007; Lodish et al., 2000). Hemopexin is a 60 kDa glycoprotein which has been shown to be mainly expressed in the hepatic parenchymal cells (Thorbecke et al., 1973). It belongs to the acute-phase protein family, whose synthesis is induced by several cytokines in response to an inflammatory event (Baumann and Gauldie, 1994). In addition, hemopexin is the circulating protein with the highest affinity for heme, and it is considered to be the major responsible for its transport (Tolosano et al., 1999). This feature has led to the belief that hemopexin prevents the body from heme-catalyzed oxidation as well as heme-bound iron loss, thus protecting against inflammation and liver fibrosis (Tolosano et al., 1999). Our results in the training group showed a significantly increased serum concentration of hemopexin in cirrhotic rats as compared to control animals.

The results obtained in the validation group confirmed SIPA1L1 as a biomarker but failed to demonstrate that hemopexin is an appropriate indicator of cirrhosis in rats. In fact, assessment of the serum concentration of hemopexin by ELISA in the different groups of CCl_4_-treated rats, including cirrhotic animals, did not show significantly different values as compared to control animals. Accordingly, the diagnostic accuracy of this parameter, as assessed by the ROC curve, failed to show statistical significance. By contrast SIPA1L1 does demonstrate excellent diagnostic accuracy for fibrosis, being more remarkable when evaluating samples from rats with mild/moderate fibrosis (data not shown). In fact, the serum concentrations of SIPA1L1 in this group of animals showed an approximately 40% reduction in comparison to healthy animals. Therefore, a reduction of SIPA1L1 serum concentration could detect the early fibrotic subjects who are prone to develop more severe complications and allow prompt therapeutic interventions.

Despite no studies having described the potential role that this protein may have in liver fibrosis, a previous investigation (Tsai et al., 2007) demonstrated that Wnt signaling affects the phosphorylation and stability of SIPA1L1. In particular, Wnt signaling activates casein kinase I epsilon (CKIε) (Swiatek et al., 2004), which induces SIPA1L1 phosphorylation and its degradation as well as the accumulation of β-catenin (Gao et al., 2002). Since the Wnt signaling pathway is activated during liver fibrosis in hepatic stellate cells (Miao et al., 2013) it is tentative to speculate that SIPA1L1 diminution is a consequence of the activation of the fibrogenic process. In this regard, several investigations have shown that sustained Wnt/β-catenin pathway activation is linked to the pathogenesis of different fibrotic disorders including liver fibrosis (Cheng et al., 2008, 2010; He et al., 2009; Jiang et al., 2006; Li et al., 2011; Myung et al., 2007). Therefore, these results are consistent with the hypothesis that SIPA1L1 downregulation is a surrogate marker of early fibrogenesis activation in liver disease. However, the detailed mechanism by which this phenomenon could happen still needs to be elucidated.

The current investigation took advantage of the *in vivo* model of fibrosis induction by CCl_4_ inhalation in Wistar rats to identify potential new biomarkers related to liver fibrosis progression. Using a label-free LC-MS/MS proteomic approach, we performed bioinformatics analyses to explore and visualize a protein biomarker fingerprint associated with hepatic fibrosis. Subsequently, we selected the 10 proteins with the greater discriminatory power between fibrosis or cirrhosis and the control group. Among them, hemopexin and SIPA1L1 were picked for further validation. Despite hemopexin failing to demonstrate its value as an indicator of cirrhosis, SIPA1L1 showed a clear serum diminution and a great accuracy at identifying early fibrotic stages in the validation group. This study, therefore, strengthens the importance of combining reliable experimental models with holistic proteomic approaches to uncover new biomarkers of fibrosis in liver disease.

## MATERIALS AND METHODS

### Animal studies

The study was performed in 94 male adult Wistar fibrotic/cirrhotic rats and 36 control Wistar rats (Charles-River, Saint Aubin les Elseuf, France). The design of the study was two folded: firstly, using quantitative label-free LC-MS/MS we assessed a possible correlation between fibrosis stage and serum proteomic expression in a training group of 18 rats with different degrees of fibrosis. Sixteen rats were used as the control group. At the end of the study, the animals were sacrificed by isofluorane overdose and serum samples from each rat were obtained and kept at −80°C until further analysis. Afterwards, to validate the usefulness of the biomarkers identified in the training protocol, two different quantitative enzyme immunoassays (ELISA) were performed in a validation set of 76 CCl_4_-treated rats and 20 healthy animals. Standard liver and renal function tests were also analyzed in all animals.

### Induction of fibrosis and cirrhosis in rats

Fibrosis was induced by repetitive CCl_4_ inhalation as described previously (Clària and Jiménez, 2005). Briefly, rats were fed *ad libitum* with standard chow and water containing 0.3 g/l of phenobarbital as drinking fluid. Animals were exposed to a CCl_4_ vapor atmosphere twice a week, starting at 30 s per exposure. The duration of the inhalation was increased by 30 s after the first three sessions and by 1 min after every other three sessions until it reached a plateau of 5 min. To obtain variable degrees of hepatic fibrosis, CCl_4_-treated rats were investigated between the 9th and the 40th week after starting the fibrosis-induction protocol. Control rats were studied after similar periods of phenobarbital administration alone.

### Hemodynamic studies

Systemic and portal hemodynamics was assessed in 76 CCl_4_-treated rats and 20 control rats as described elsewhere (Reichenbach et al., 2012). In short, animals were anesthetized with Inactin (50 mg/kg body weight; Sigma-Aldrich, St. Louis, MO, USA) and a catheter was implanted in the right femoral artery. Hemodynamic parameters were allowed to stabilization for 30 min, and the mean arterial pressure (MAP) and heart rate (HR) values were registered for two periods of 30 min. Each value shown in Table 3 represents the average of these two measurements. Subsequently, an abdominal incision was performed, and another catheter was placed in the portal vein through the ileocolic vein and fixed to the mesentery with cyanoacrylate glue. After verifying free blood reflux, portal pressure (PP) was registered. Splanchnic perfusion pressure (SPP) was estimated as MAP-PP. At the end of the study, animals were sacrificed and serum and liver tissue samples were obtained and kept as described in the ‘Animal studies’ section.

### Quantification of fibrosis

Four-micron-thick sections of paraffin-embedded liver were stained in 0.1% Sirius Red F3B (Sigma-Aldrich) in saturated picric acid (Sigma-Aldrich). The relative fibrosis area, expressed as a percentage of the total liver area, was evaluated by analyzing a minimum of 30 fields of Sirius Red-stained liver sections per animal. Each field was acquired at 10× magnification (Eclipse E600; Nikon, Kawasaki, Kanagawa, Japan), and the images obtained were analyzed using ImageJ software (version 1.47; NIH). According to the percentage of fibrosis area the histological lesion was classified as mild/moderate fibrosis (<6%), severe fibrosis (6-11%) or cirrhosis (>11%).

### Label-free LC-MS/MS proteomics and bioinformatic analysis of the results

Pooled samples from rats with mild (*n*=6) or severe fibrosis (*n*=6) and cirrhosis (*n*=6) were fractionated using Seppro IgY7 LC10 columns (Genway Biotech Inc., USA) in order to remove the seven most abundant serum proteins (albumin, IgG, fibrinogen, alpha-1-antitrypsin, haptoglobin, transferrin and IgM). In addition, three pools of samples from 5, 5 and 6 control rats were also prepared and fractionated as described. After fractionation, 20 μg of total protein from each pool were digested. The organic solvent of the sample was evaporated in a Savant SpeedVac concentrator (Thermo Fisher Scientific, Waltham, MA, USA), and the pellet was redissolved in 20 μl 8 M urea/0.4 M ammonium bicarbonate. Proteins were reduced after incubation with 2 μl of 45 mM dithiothreitol (DTT) and alkylated after incubation with 2 μl of 100 mM iodoacetamide. Then, the samples were digested with 2 μg of Lys C 0.1 mg/ml for 4 h and 2 mg of trypsin 0.1 mg/ml for 12 h. Digested proteins were evaporated and the peptides were dissolved in 5 μl 70% formic acid+25 μl 0.1% trifluoroacetic. Five microliters of each experimental sample at the same peptide concentration were injected on a LTQ Orbitrap Velos (Thermo Fisher Scientific) through a nanoAcquity ultra performance liquid chromatography (UPLC) (Waters Corporation, Mildford, MA, USA) system equipped with a Water Symmetry C18 column (180 μm×20 mm). Trapping was done at 15 µl/min in the presence of 0.1% formic acid for 1 min. Peptide separation was performed with a linear gradient over 90 min at a flow rate of 300 nl/min. Mass spectra were acquired using a maximum injection time of 900 ms followed by three data dependant MS/MS acquisitions in the ion trap (with a precursor ions threshold of >3000). The total cycle time for both MS and MS/MS acquisition was 2.4 s. Peaks targeted for MS/MS fragmentation by collision-induced dissociation (CID) were first isolated with a 2 Da window followed by normalized collision energy of 35%. Samples of each experimental condition were injected in triplicate. The data were processed using the Progenesis LC-MS (Nonlinear Dynamics, LLC), R (http://www.r-project.org), Mascot (Matrix Science) and IPA programs (Ingenuity Systems). Briefly, the original files with the results obtained from the LTQ Orbitrap were imported into Progenesis, and the spectra of the different experimental conditions were aligned to minimize variability in chromatographic retention times. No adjustments were necessary in the m/z dimension due to the high mass accuracy of the spectrometer (<3 ppm). Features within retention time ranges of 0-25 min and 110-120 min were filtered out, as were features with a charge≥+8. The .mgf files generated in Progenesis were analyzed using the Mascot algorithm based on SwissProt rat data. The following parameters were set for the Mascot algorithm: trypsin enzyme, carbamidomethyl and oxidation for variable modifications, monoisotopic for mass values, unrestricted protein mass, ±20 ppm for peptide mass tolerance, ±0.6 Da for fragment mass tolerance, +7 for charge, 3 for max missed cleavages and ESI-TRAP for instrument type. We considered a protein identified when Mascot listed it as significant and more than two unique peptides matched the same protein. Search hits were assigned to corresponding features using Progenesis LCMS software. The data obtained were normalized by the method of robust quantile normalization and statistical differences were assessed using linear models and the moderate *t*-test (LIMMA package, Bioconductor) (Ritchie et al., 2015). Subsequently, diseases, biological functions and canonical pathways were studied using the Ingenuity Pathways Knowledge Base (Ingenuity System Inc, USA). The proteomic results were compared with global molecular networks using the Fisher's Exact Test. The resulting *P*-values were adjusted for multiple comparisons using the Benjamini and Hochberg's method to control the false discovery rate. After multitest adjustment, differences were considered to be significant at a *P*-value less than 0.05. For the canonical pathway analysis, the significance of the association was additionally measured considering the ratio of the number of significant targets that map to the pathway divided by the total number of targets that are included in the canonical pathway. Multivariate data analysis was performed using R. Finally, the GeneCards database (http://www.genecards.org/) was used to check homologies between the rat and human genes encoding for the proteins which were statistically significant among all the different groups.

### Biomarkers assessment in the validation group

The proteomic analysis resulted in the identification of two circulating proteins, hemopexin and SIPA1L1 as the most promising biomarkers of liver fibrosis in CCl_4_-treated rats. Next, the circulating levels of SIPA1L1 were measured by ELISA in an additional group of 76 CCl_4_-treated rats (25 rats with mild fibrosis, 25 rats with severe fibrosis and 26 rats with cirrhosis). Hemopexin was also determined by ELISA in 47 of them (15 rats with mild fibrosis, 15 rats with severe fibrosis and 17 rats with cirrhosis). 20 control rats were included in the SIPA1L1 validation protocol, 15 of which were also used in the hemopexin study. Hemopexin was measured according to the manufacturer's protocol (USCN Life Science Inc, Wuhan, China) whereas SIPA1L1 reagents were obtained from EIAab (Wuhan, China), and minor modifications of the manufacturer's instructions were made. Briefly, wells were incubated with either 100 μl of serum, standard dilution series or standard diluent (blank) for 2 h at 37°C. After liquid removal, 100 μl of biotin-conjugated polyclonal antibody were then added at a dilution of 1:100 and allowed to bind for 1 h at 37°C. Subsequently, well plates were washed three times, and avidin conjugated to horseradish peroxidase (1:100 dilution) was incubated for 1 h at 37°C. The aspiration and wash processes were repeated five times and 90 μl of substrate solution was added to each well and allowed to bind for 20 min at 37°C, followed by the addition of 50 μl of 1 M H_2_SO_4_. Well plates were immediately read at 450 nm. The optical density value obtained from the sample diluent was subtracted from each standard determination. Subsequently, the standard curve was generated. All were measured in duplicate. In addition, the SIPA1L1 ELISA kit assay was evaluated and obtained satisfactory results in the dilution linearity test (range between 81.2% and 118.4%) as well as in the recovery test (range between 89.3% and 109.8%). Furthermore, the intra-assay (well to well) and inter-assay (plate to plate) coefficient of variation (CV) were also determined, being of 6.09% and 7.51%, respectively.

### Measurements and statistical analysis

Standard parameters of renal and liver function were measured using the BS-200E Chemistry Analyzer (Mindray Medical International Ltd, Shenzhen, China). The diagnostic accuracy of the different methods was analyzed by constructing receiver-operating characteristic (ROC) curves and calculating the area under the ROC curve (AUROC). GraphPad Prism 5 (GraphPad Software, Inc., San Diego, CA) was used for the analysis of the results, the development of the ROC curve and the selection of the optimal SIPA1L1 cutoff value. Statistical analysis were performed by one-way analysis of variance (ANOVA) with Newman–Keuls post hoc test or Kruskal–Wallis test with the Dunn post hoc test when appropriate, based on the results obtained from the D'Agostino and Pearson omnibus normality test. Results are expressed as mean±s.e.m. and *P*-values less than 0.05 were considered significant.

### Ethical approval

The study was approved and performed according to the criteria of the investigation and ethics committee of the University of Barcelona [Comité Ético de Experimentación Animal (CEEA)]. The authors state that they have obtained appropriate institutional review board approval or have followed the principles outlined in the Declaration of Helsinki for all human or animal experimental investigations.
